# Health Risks Associated with Informal Electronic Waste Recycling in Africa: A Systematic Review

**DOI:** 10.3390/ijerph192114278

**Published:** 2022-11-01

**Authors:** Ibrahim Issah, John Arko-Mensah, Thomas P. Agyekum, Duah Dwomoh, Julius N. Fobil

**Affiliations:** 1 Department of Biological, Environmental and Occupational Health Sciences, School of Public Health, College of Health Sciences, University of Ghana, Legon, Accra 00233, Ghana; 2 Department of Occupational and Environmental Health and Safety, School of Public Health, College of Health Sciences, Kwame Nkrumah University of Science and Technology, Kumasi 00233, Ghana; 3 Department of Biostatistics, School of Public Health, College of Health Sciences, University of Ghana, Legon, Accra 00233, Ghana

**Keywords:** e-waste, Africa, health outcome

## Abstract

Informal electronic waste (e-waste) recycling in Africa has become a major public health concern. This review examined studies that report on the association between e-waste exposure and adverse human health outcomes in Africa. The review was conducted following the updated version of the Preferred Items for Systematic Review and Meta-analysis (PRISMA 2020) statement checklist. We included papers that were original peer-reviewed epidemiological studies and conference papers, written in English, and reported on e-waste exposure among human populations and any health-related outcome in the context of Africa. Our results from the evaluation of 17 studies found an association between informal e-waste recycling methods and musculoskeletal disease (MSD) symptoms and physical injuries such as back pains, lacerations, eye problems, skin burns, and noise-induced hearing loss (NIHL). In addition, the generation and release of particulate matter (PM) of various sizes, and toxic and essential metals such as cadmium (Cd), lead (Pb), zinc (Zn), etc., during the recycling process are associated with adverse systemic intermediate health outcomes including cardiopulmonary function and DNA damage. This systematic review concludes that the methods used by e-waste recyclers in Africa expose them to increased risk of adverse health outcomes. However, there is a need for more rigorous research that moves past single pollutant analysis.

## 1. Introduction

Informal electronic waste (e-waste) recycling uses primitive techniques, without or with very little technology to retrieve valuable materials such as copper, silver, tin, and gold from old electrical and electronic products [1]. These informal recycling activities, such as open burning, which persist in developing countries, have led to the emission of pollutants of public health concern into the ambient environment [2,3,4,5]. E-waste workers and people living near e-waste sites are exposed to elevated levels of these pollutants compared to the general population. Extant literature exists to show evidence of the body burden of metals [5,6,7,8] and organic pollutants [3,9,10] due to e-waste recycling activities.

Informal e-waste recycling in African countries has become a major source of toxic pollutants [11,12]. Before the demolishing of the Agbogbloshie e-waste recycling site in central Accra by the government of Ghana in July 2021 [13], it was regarded as one of the largest, busiest, polluted, and harshest informal e-waste recycling sites worldwide [14,15]. In addition, the Alaba International electronic market and Ikeja Computer Village in Nigeria are major destinations of shipped e-waste from Europe and the USA [16,17]. Previous studies have reported elevated levels of toxic metals, polycyclic aromatic hydrocarbons (PAHs), volatile organic compounds (VOCs), and particulate matter (PM) among e-waste workers compared to the general population [2,6,8,9,10,18].

E-waste exposure has been reported to be associated with adverse health outcomes such as DNA damage [19], decreased lung function [20,21], change in thyroid function, adverse neonatal outcomes, changes in temperament and behaviour [1], and a myriad of chronic disease risks, including cancer [22,23]. A recent systematic review update on the health consequences of e-waste exposure by Parvez et al. (2021) [24] found 70 studies that concluded that “the existence of various toxic chemicals in e-waste recycling areas impose plausible adverse health outcomes”. Almost all these studies (*n* = 66/70) were conducted in China, while only one study conducted by Burns et al. (2016) [25] was focused on Africa (Ghana). Drawing inferences from these studies and extrapolating them to e-waste-exposed populations in Africa could be invalid because of the vastly different social, demographic, and host genetics. In addition, these previous reviews on the health consequences of e-waste exposure conceptualized the health outcomes as biological responses (e.g., disturbance in thyroid hormone, DNA damage, etc.) associated with chemical exposures without paying attention to physical and psychological outcomes (e.g., cuts, lacerations, and perceived stress) associated with the crude recycling methods mostly encountered by e-waste workers in Africa [1,24]. Grant et al. [1] further concluded that there were limited studies on the effects of e-waste on health in developing countries in Africa. Since then, researchers have shown an increased interest in examining the health risks of informal e-waste recycling in African countries including Ghana [26,27,28,29], Nigeria [16,30], Benin, Burkina Faso, the Ivory Coast, Mali, and Senegal [31,32]. However, there is a current paucity of evidence-based literature describing the health impacts of e-waste exposure in Africa where African nations continue to be major recipients of e-waste shipped from developed nations. Therefore, before proceeding with recommendations for policy makers in Africa on e-waste management programs, it is necessary to understand the burden on adverse health of e-waste recycling in Africa. The present review is set to fill this gap in the literature by providing a summary of the evidence of the association between e-waste exposure and adverse human health outcomes in Africa to guide policies to ensure the health and safety of vulnerable populations.

## 2. Methods

This review was conducted according to the updated version of the Preferred Items for Systematic Review and Meta-analysis (PRISMA 2020) statement checklist [33] (Supplementary Table S1).

### 2.1. Eligibility Criteria

Published manuscripts were included for this review if they were original peer-reviewed epidemiological studies and conference papers written in English, and reported on e-waste exposure among human populations and any health-related outcome in the context of Africa. We excluded studies that were not original (e.g., reviews, abstracts, editorials, correspondence, reports, book chapters, preface, commentary), not written in English, that did not measure any health-related outcomes concerning e-waste exposure, that reported outcomes in plants or animals, and that were not conducted in Africa.

### 2.2. Information Sources and Search Strategy

We conducted a search in two major electronic databases: Scopus and MEDLINE via EBSCOhost, for published peer-reviewed journal articles. The searches were not restricted to a specific period. The search terms used included (“Electronic waste” OR “E-waste” OR “Waste electrical and electronic equipment” OR “WEEE”) AND (“Health” OR “Respiratory health” OR “injuries” OR “Cardiovascular health” OR “heart rate variability” OR “blood pressure” OR “DNA” OR “musculoskeletal disorders” OR “stress” OR “hearing disorders”) AND (“Africa” OR “Sub-Saharan Africa” OR “developing countries” OR “Cameroon” OR “Chad” OR “Congo” OR “Democratic Republic of Congo” OR “Congo, Demographic Republic” OR “Congo, Republic” OR “Equatorial Guinea” OR “Gabon” OR “Burundi” OR “Djibouti” OR “Eritrea” OR “Ethiopia” OR “Kenya” OR “Rwanda” OR “Somalia” OR “Sudan” OR “Tanzania” OR “Uganda” OR “Angola” OR “Botswana” OR “Lesotho” OR “Malawi” OR “Mozambique” OR “Namibia” OR “Swaziland” OR “Zambia” OR “Zimbabwe” OR “Benin” OR “Burkina Faso” OR “Cape Verde” OR “Cote D’ivoire” OR “Gambia” OR “Ghana” OR “Guinea” OR “Guinea-Bissau” OR “Liberia” OR “Mali” OR “Mauritania” OR “Niger” OR “Nigeria” OR “Senegal” OR “Sierra Leone” OR “Togo” OR “South Sudan” OR “Madagascar” OR “Comoros” OR “Mauritius” OR “Sao Tome and Principe” OR “Seychelles” OR “South Africa” OR “Algeria” OR “Egypt” OR “Libya” OR “Morocco” OR “Tunisia”). The reproducible full search strategies and results for all databases are presented in Supplementary Table S2. The search was conducted on 8 July 2022 for both the two databases, and updated on 9 September 2022 by rerunning the search strategies using the same databases.

### 2.3. Selection Process

**Figure 1:**
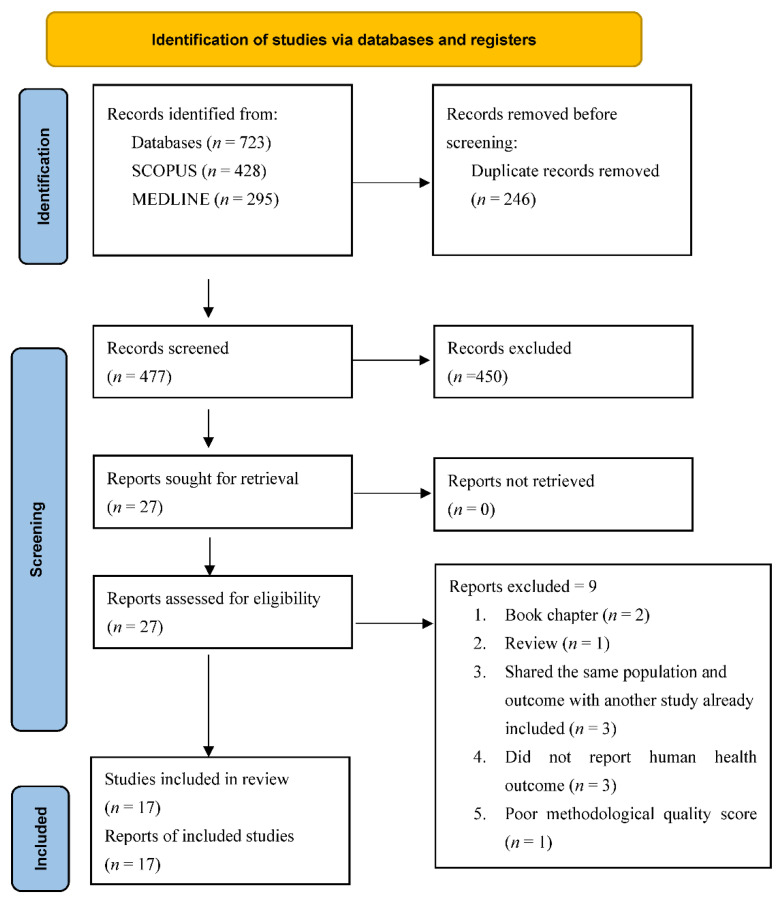
PRISMA flow chart illustrating the process of selecting the studies included in the review.

Following the search, all identified citations were collated and uploaded into EndNote version 8 (Clarivate Analytics, Pennsylvania, PA, USA), and duplicates were removed using the EndNote’s duplicate identification strategy and manual processes. Two reviewers (II and TPA) independently screened titles and abstracts of each citation against the inclusion criteria for the review. We resolved discrepancies through consensus, and all relevant sources were retrieved in full. The full texts of selected citations were assessed in detail against the inclusion criteria. The results of the search and the study inclusion process are presented in an updated PRISMA 2020 flow diagram [33] (Figure 1).

### 2.4. Data Collection Process

A data extraction form was adapted from Parvez et al. (2021) [24] to collect the relevant information from each included study. We developed a standardized protocol to include the following study characteristics: name of author and year of publication, study design, location study was conducted, sample size, type of pollutant(s) assessed, health outcomes measured, and effect sizes of the association between the exposures and health outcomes. Two reviewers (II and TPA) used the protocol to extract data independently, and any discrepancies were resolved through discussion with a third reviewer (JA-M).

### 2.5. Study Risk of Bias Assessment

Two reviewers (II and TPA) independently assessed the methodological quality of all included studies using the Joanna Brigg’s Institute (JBI) critical appraisal checklists for analytical cross-sectional and cohort studies [34] (Supplementary Tables S6 and S7). The checklists consist of 8 and 11 major items for the cross-sectional and cohort studies, respectively. Each item was awarded a score of one for a “yes” response and zero for a “no”, “not clear” or “not applicable” response. Studies were considered high quality if they scored greater than the median score of 4 for the cross-sectional studies and greater than 5 for the cohort studies. Any disagreements were resolved through discussion between the two reviewers.

## 3. Results

### 3.1. Study Selection

The initial search of the two databases and hand-curated literature yielded 723 publications, of which 246 duplicates were identified and removed. The remaining 477 publication’s titles and abstracts were screened against the inclusion criteria. At this stage, 450 publications were excluded, allowing 27 to advance into the full-text screening phase. After the full-text screening, 10 publications were excluded with reasons (Supplementary Table S5), and the remaining 17 publications were deemed eligible for inclusion in the review. Figure 1 provides a PRISMA flowchart representing the process of study selection for this review.

### 3.2. Study Characteristics

The review identified 17 eligible studies for inclusion with a total citation frequency of 355. The majority of the studies were conducted in Ghana (*n* = 10), followed by Nigeria (*n* = 4), Senegal (*n* = 1), Benin (*n* = 1), and five West African French-speaking countries: Benin, Burkina Faso, Ivory Coast, Mali, and Senegal (*n* = 1). All the studies done in Ghana were conducted at the Agbogbloshie e-waste recycling site. The majority of the studies (14 out of 17) used a cross-sectional design, and the remaining three studies [21,35,36] used a cohort study design. Thirteen of the included studies were conducted among male e-waste workers, and only three studies [30,32,37] included female participants. Of the 17 studies, eight did not include a reference population, and the sample size ranged between 45 [38] and 740 [32].

**Figure 2:**
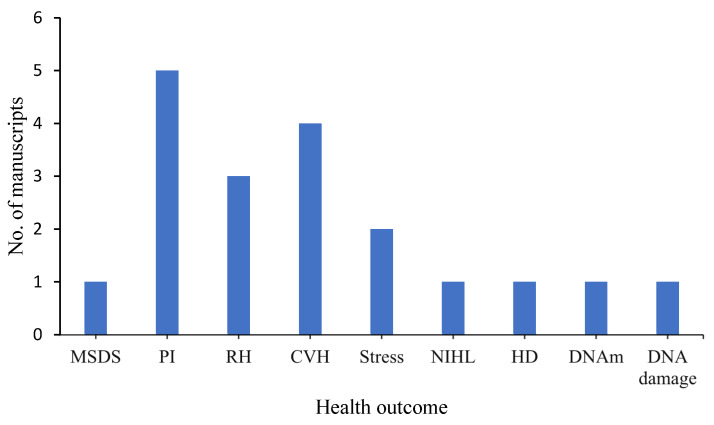
Summary of manuscripts that reported on various health outcomes. Abbreviations: MSDS—musculoskeletal disease symptoms; PI—physical injuries (cuts, lacerations, etc.); RH—respiratory health; CVH—cardiovascular health; NIHL—noise-induced hearing loss; HD—hepatic disease; DNAm—DNA methylation. NB: Some manuscripts reported more than one health outcome.

The included studies assessed a wide range of health indicators including musculoskeletal diseases and symptoms [26], observed and self-reported physical injuries, and respiratory symptoms [27,30,31,37,38,39], markers of cardiovascular health [25,35,36,40], perceived stress [32], markers of respiratory health [21], noise-induced hearing loss [28], markers of hepatic damage [41], epigenetic modification (DNA methylation) [42], and markers of DNA damage [16] (Figure 2). Ten out of the seventeen studies [25,26,27,30,31,32,37,38,39,41] used the e-waste recycling methods such as dismantling and burning as the main exposures, four studies [16,28,40,42] measured metals in the blood as the main exposure biomarkers, and three studies measured the breathing-zone PM as the main exposures [21,35,36] (Table 1).

### 3.3. Quality of Evidence from the Included Studies

The methodological quality assessment of the included studies was conducted using the JBI Critical Appraisal Checklist for analytical cross-sectional and cohort studies (Supplementary Tables S3 and S4). Twelve out of thirteen of the included cross-sectional studies had high-quality assessment scores of 5–7, two studies attained an average score of 4, while one study attained a poor quality assessment score of 2, and therefore was excluded at the data extraction phase. Regarding the cohort studies, all three studies attained high-quality assessment scores of 9–10, and therefore were all included for analysis.

### 3.4. Narrative Synthesis of Study Outcomes

Six of the included studies in this review reported on musculoskeletal diseases (MSDs) symptoms, self-reported health symptoms, and physical injuries associated with e-waste recycling activities, of which four were conducted in Ghana [26,27,37,39]; the remaining two studies were done in Benin [38] and Nigeria [30]. In general, the crude recycling methods such as manual dismantling, collecting, and open burning of e-waste were associated with an increased risk of health symptoms. For example, Acquah et al. (2021) [26] examined the prevalence and intensity of self-reported musculoskeletal disorder (MSD) symptoms among e-waste workers (*n* = 176) at Agbogbloshie, with 41 non-e-waste workers as a reference group. The results of their study showed that e-waste collectors and dismantlers reported a higher number of body parts with musculoskeletal discomforts compared to the reference population. In addition, a 1-week discomfort prevalence was highest among e-waste collectors (91.8%), followed by dismantlers (89%), and burners (81.7%), with the reference population reporting the lowest prevalence (70.7%) of discomfort [26]. Using spatial assessment and analysis, Adusei et al. (2020) [27] physically examined, characterized, and enumerated physical injuries among e-waste workers (*n* = 112) at the Agbogbloshie site. The study identified scars as the most common (93.6%) skin condition of the e-waste workers. Further analysis revealed that e-waste dismantlers had a higher mix of scars, lacerations, and abrasions, with a 23.1% prevalence of burns among the study participants [27]. Similarly, Armah et al. (2019) [37] set out to examine differences in self-reported experiences in disease symptoms among two different categories of e-waste exposed populations (Agbogbloshie resident e-waste workers (*n* = 140) and non-e-waste workers (*n* = 60), and a community in Winneba in the central region as the control population with no e-waste exposure (*n* = 60)). Resident e-waste workers were 102% and 84% more likely to report that they experience eye problems and skin burns, respectively, than their resident non-e-waste counterparts. However, participants from the control population were 64% and 54% less likely to report eye problems and skin burns, respectively, compared to the resident non-e-waste workers [37]. In addition, resident e-waste workers reported more breathing difficulty (odds ratio (OR) = 3.30, confidence interval (CI): 1.733–6.267) compared with their counterparts who were resident non-e-waste workers. In contrast, the control population were less likely to report breathing difficulty (OR = 0.24, *p* < 0.01) compared with their counterparts who were resident non-e-waste workers [37]. In one study [39], injury experiences, noise exposure, and stress risk factors were evaluated among male e-waste workers (*n* = 46) in Agbogbloshie [39]. E-waste workers experienced an average ± SD of 9.9 ± 9.6 injuries per person and an average ± SD noise level of 78.8 ± 5.9 dBA with 15% of time-weighted average (TWA) exposures exceeding the recommended 85 dBA occupational exposure limit. The most-reported injuries were lacerations to the hands. Findings from a multivariable Poisson regression showed that higher perceived stress levels, not using gloves, and higher perceived noise exposure frequency was significantly associated with a higher number of injuries [39]. The Beninese study [38], reported a high prevalence of injuries (88.9%), itchy skin (45.2%), melena (16.3%), and haematuria (14.2%) among individuals (*n* = 45) involved in e-waste recycling, with the number of hours worked per day associated with haematuria, itchy skin, and airway obstruction. Similarly, Ohajinwa et al. (2018) [30] documented the prevalence and injury patterns among informal e-waste workers (*n* = 279) in Nigeria. The results of their study showed a high prevalence (68%) of injuries, which were mainly cuts to the hands and fingers (59%), and mostly caused by sharp objects (82%) [30].

Four studies [25,35,36,40] examined cardiovascular function associated with e-waste recycling work. In one study [25], the investigators focused on noise exposure and heart rate among e-waste workers (*n* = 57). In total, 43.5% of the participants had noise exposures that exceeded the acceptable occupational limits of 85 dBA, and 45% had noise exposure that exceeded the community acceptable limit of 70 dBA. A mixed-effects linear regression model indicated that a 1 dBA increase in noise exposure was associated with a 0.17 increase in heart rate (*p* = 0.01) [25]. Two longitudinal studies [35,36] evaluated particulate matter (PM) as the main exposure associated with heart rate, heart rate variability (HRV), and blood pressure related to e-waste work. Elevated concentrations of breathing zone PM_2.5_ and PM_10_ were observed in e-waste recyclers compared to controls [35,36]. The authors of one study reported higher systolic blood pressure among the control population (mean ± SD = 128.57 ± 2.12 vs. 123.06 ± 1.03 mmHg). However, exposure to 1 μg/m^3^ of PM_2.5_ was associated with an increased heart rate (HR) among the e-waste recyclers [36]. Similarly, Amoabeng Nti et al. (2021) [35] reported that a 10 μg/m^3^ increase in the concentrations of PM_2.5_, PM_10–2.5_, and PM_10_ in personal air was associated with reduced HRV indices and increased resting HR. A Nigerian study reported a higher risk of cardiovascular health among e-waste workers in Benin city, Nigeria (*n* = 63) than in a reference population in the Ugbowo community (*n* = 41) with no e-waste exposure [40]. The authors reported that lipid profile and atherogenic indices such as total cholesterol, low density lipoprotein (LDL) cholesterol, atherogenic coefficient (AC), and Castelli risk indices (CRI-1 and CRI-11) were significantly increased in the e-waste exposed participants compared to the non-exposed group. The high levels of cadmium observed in the e-waste workers correlated positively with total cholesterol and LDL-cholesterol [40].

Amoabeng Nti et al. (2020) [21] conducted a longitudinal study across three seasons of the year (dry, rainy and harmattan) to determine the association between concentrations of personal PM (2.5, 2.5–10 and 10 µm) and lung function of informal e-waste workers at Agbogbloshie (*n* = 142) and a reference population in Madina-Zongo (*n* = 64) with no e-waste exposure. The authors assessed lung function using the following lung function parameters; forced vital capacity (FVC), forced expiratory volume in one second (FEV1), a ratio of forced expiratory volume in one second and forced vital capacity (FEV1/FVC), peaked expiratory flow (PEF), and forced expiratory flow 25–75 (FEF25–75). Generally, PM (2.5, 2.5–10, and 10 µm) concentrations were significantly higher among the e-waste workers compared to the control population across all seasons. Further analysis showed that a 10 µg increase in PM (2.5, 2.5–10, and 10 µm) was associated with decreases in lung function, as measured by PEF and FEF 25–75, by 13.3% (β = −3.133; 95% CI: −0.243, −0.022) and 26.6% (β = −0.266; 95% CI: −0.437, 0.094), respectively. Similarly, the respiratory health of e-waste workers (*n* = 178) across 17 sites distributed in six areas in Dakar, Senegal was evaluated [31]. A spirometry-defined respiratory disease revealed 7.11% cases of asthma, 21.65% cases of obstructive COPD-like syndrome, and 7.22% cases of restrictive syndromes among the e-waste workers [31].

In three studies, metals exposure associated with e-waste recycling were investigated as the main toxic agent associated with hearing loss [28] and DNA damage [16,42]. In one of the studies [28], the authors measured the levels of toxic (cadmium (Cd), lead (Pb), arsenic (As), and total mercury (Hg)) and essential elements (selenium (Se), manganese (Mn), copper (Cu), iron (Fe), and zinc (Zn)) in blood, and administered audiometric tests on e-waste workers (*n* = 58). The average levels of Pb (mean = 9.72 µg/dL), Cd (mean = 2.98 ppb), Mn (mean = 12.6 µg/L), and Se (mean = 163.6 µg/L) were all higher than the US adults average [28]. Even though the toxic metals (Pb, Cd, and Hg) did not significantly predict noise-induced hearing loss (NIHL), participants who lived at the e-waste recycling site (Agbogbloshie) for longer periods were significantly associated with worse hearing thresholds at 4 and 6 kHz. Similarly, Issah et al. (2022) [42], assessed the levels of blood Pb, Cd, Zn, Se, and Mn and global (LINE-1) DNA methylation among e-waste workers in Agbogbloshie (*n* = 100) and a control group in Madina-Zongo (*n* = 51) with no e-waste exposure. E-waste workers had a higher concentration of Pb in their blood than their counterparts in the control group (geometric mean, Pb = 79.6 vs. 37.7 µg/L, *p* < 0.001). In contrast, Cd, Se, and Mn were significantly higher in the control group than in the e-waste worker’s group. Global DNA methylation levels did not differ significantly between the e-waste workers and the control group (mean ± SD = 85.1 ± 1.3 vs. 85.2 ± 1.1, *p* = 0.785). However, an elevated concentration of blood Zn was associated with a significant decrement in global DNA methylation levels (βZn = −0.912; 95% CI, −1.512, −0.306; *p* = 0.003) [42]. In teenage e-waste scavengers in Nigeria, blood Pb, Cd, Cr, and Ni were significantly associated with micronuclei frequency in buccal exfoliated cells [16].

A Nigerian study quantified and compared the serum activities of liver enzymes between e-waste workers (*n* = 63) and a reference group (*n* = 41) with no e-waste exposure to assess the risk of liver damage in e-waste workers [41]. The results showed significantly raised activities of enzymatic biomarkers of liver damage (alanine aminotransferase (ALT), aspartate aminotransferase (AST), alkaline phosphatase (ALP), and gamma glutamyltransferase (GGT)) in the e-waste worker group compared with the reference population. In addition, serum alpha-fetoprotein (AFP), a biomarker of liver cancer risk, was significantly higher in the e-waste workers compared to the control group (3.56 ± 0.34 vs. 2.14 ± 0.80 ng/mL; *p* < 0.045) [41].

In e-waste workers (*n* = 740) across five French-speaking West African countries (Benin, Burkina Faso, the Ivory Coast, Mali, and Senegal), a high prevalence (76.76%) of perceived stress was reported among the e-waste workers [32]. Factors associated with the high perceived stress level in this study participants included insufficient income, number of working days per week, perceived violence at work, and the interference of work with family responsibilities or leisure [32]

## 4. Discussion

To the best of our knowledge, this systematic review is the first to assess the health risks associated with informal e-waste recycling in Africa. The review identified 17 studies that examined a wide range of health outcomes associated with e-waste exposure. A majority (59%) of the included studies were conducted at the Agbogbloshie e-waste site in Ghana. Findings from this review suggest an association between informal e-waste recycling methods and musculoskeletal disease symptoms and physical injuries such as lacerations, eye problems, skin burns, and noise-induced hearing loss (NIHL), among others. In addition, the generation and release of PM of various sizes, and toxic and essential metals such as Cd, Pb, Zn, etc., during the recycling process are associated with adverse intermediate health outcomes including increased heart rate, reduced heart rate variability, reduced lung function, increased risk of liver, and DNA damage.

This current review found that informal e-waste recycling methods such as collecting and manual dismantling of waste materials are associated with higher discomfort and pain to the lower back, shoulders, and knees among e-waste workers compared to non-e-waste workers. In addition, physical injuries such as cuts, lacerations, skin burns, and eye problems are associated with informal e-waste recycling methods. These findings corroborate with other studies conducted among informal e-waste workers in other lower-middle income countries in Latin America such as Chile [43], and Thailand [44]. In the Chilean study, the authors reported that the most common injuries to e-waste workers are cuts and lacerations to the hands/fingers and legs. In addition, participants complained of MSD symptoms such as pain in their hands or wrists after working with e-waste, and muscle soreness from sitting in the same position for long periods [43]. The prevalence of injuries and MSD symptoms reported by informal e-waste workers could be attributed to the use of locally made simple tools such as a hammer and chisel, and occasionally screwdrivers and spanners, to break apart e-waste to separate the different metals [45], and inconvenient postures and lifting of heavy loads during the recycling process. E-waste workers perform these activities with little or no use of personal protective equipment (PPE) [43,46,47], and this could further explain the higher prevalence of injuries and MSD symptoms reported in this review. Similarly, MSDs have been reported in other informal occupational groups including mining workers [48], plastic manufacturing workers [49], and handicraft workers [50].

Moreover, the review found evidence of the association between e-waste recycling-related PM exposure and increased HR, decreased HRV, and reduced lung function parameters (PEF and FEF 25–75). These studies were longitudinally designed and measured PM and cardiopulmonary intermediate health outcomes over the three seasons in Ghana (dry, rainy, and harmattan). All three studies controlled for the confounding effects of other important variables during analysis such as smoking habits, alcohol consumption, and age, among others. These studies, however, were limited by a lack of analysis of the chemical composition of PM, and a lack of control for co-pollutants such as metals and persistent organic pollutants (POPs). There is a paucity of information regarding PM exposure and cardiovascular and respiratory health outcomes among e-waste workers. However, researchers in China have reported associations between PM and metals, and cardiovascular [51,52,53], and respiratory [54,55,56] health outcomes among schoolchildren living in an e-waste exposed towns. These studies reported higher pollutants exposure in the exposed children than in the controls, and therefore could explain the link between e-waste and adverse cardiopulmonary outcomes among the study population. Because fine particulate matter (PM_2.5_) can penetrate the alveoli, components may escape from these respirable particles and enter the circulation to influence important circulatory parameters that are risk factors for adverse cardiovascular and respiratory events [57]. Many studies in the occupational air pollution literature also support the association between PM and cardiopulmonary health. For example, a systematic review of occupational PM exposure and CVDs found a strong link between PM and reduced HRV [58]. Similarly, a review by Lawin et al. (2018) [59] among commercial drivers found some evidence of occupational air pollution and adverse health outcomes, albeit some contradictory findings.

Across multiple studies, we found associations between the effects of e-waste exposure and DNA damage [16], DNA methylation [42], and increased enzymatic biomarkers of liver damage [41]. Metals exposures from e-waste recycling can damage DNA by increasing the rate of reactive oxygen species (ROS) generation within the tissue, causing oxidative stress and disruption of cellular components and structures [60]. In addition, previous studies have reported the association between toxic metals and liver damage evidenced by the increased activities of liver enzymes including aspartate aminotransferase (AST), alanine transaminase (ALT), and alkaline phosphatase (ALP) [61].

One of the strengths of this review is that, for the first time in the e-waste exposure literature, three longitudinal studies were included to assess personal PM exposure and cardiopulmonary health outcomes across three seasons using objective biomarkers of effect measurements. Even though these studies did not consider analysis based on dose–response relationships, the authors identified and controlled for important confounders. However, we acknowledge that this review is not without limitations.

First, eight studies included in this review did not include a reference population; therefore, we are unable to conclude that the associations observed are related to the e-waste recycling activities. In addition, the sample sizes of all included studies were small, and this is an already existing concern in the literature. Second, a good number of the included studies (7/17) measured outcomes such as injury frequency using self-reporting, which could affect the quality of data due to recall bias. The majority of the studies (14/17) were cross-sectional studies, hence the results are merely associations. Third, studies did not consider the measurements and analysis of cumulative and interactive effects of exposure to chemical mixtures, possibly owing to a high cost for multiple measurements, hence we are unable to account for the effects of unmeasured exposures. Fourth, the health effects of e-waste exposure on vulnerable populations such as pregnant women and children are lacking in the African region.

## 5. Conclusions

Despite the limitations outlined, the results of this review provide a summary of evidence of the association between e-waste recycling methods used in Africa and the risk of physical injuries, MSD symptoms, and NIHL. It is also shown in this review that PM and toxic metals are pollutants of concern to populations exposed to e-waste, and are linked to some systemic intermediate health outcomes including pulmonary function, cardiovascular function, liver function, and DNA damage. We hope this review report can provide useful insights for policy makers to provide appropriate recycling strategies that will consider the benefits of the processes while ensuring the health and safety of populations that depend on informal e-waste recycling for their livelihoods and survival. However, there is a need for more rigorous research that moves past single pollutant analysis to consider the measurements and analysis of cumulative and interactive effects of exposure to chemical mixtures associated with e-waste recycling.

## Figures and Tables

**Table 1:** Characteristics of included studies.

Author (Year)	Citation Frequency	Study Design	Exposed Population	Control Population	Chemical Exposure	Health Outcome	Statistical Method	Key Findings
Acquah et al. (2021) [26]	10	Cross-sectional	176 e-waste workers, Ghana	41 non-e-waste workers	NA	Self-reported musculoskeletal disorder (MSD) symptoms	Poisson regression	Comparing across body parts, discomfort prevalence for (e-waste workers vs. the reference group) was highest in the lower back (65.9% vs. 51.2%), followed by the shoulders (37.5% vs. 31.7%), knees (37.5% vs. 19.5%), lower legs (26.7% vs. 14.6%), upper arms (28.4% vs. 2.4%), and neck (26.1% vs. 22.0%).
Adusei et al. (2020) [27]	10	Cross-sectional	112 e-waste workers, Ghana	None	NA	Physical injuries	Pearson’s chi-square and ANOVA	96.2% of all the study subjects had cuts, the dismantlers had a higher mix of scars, lacerations and abrasions. Abrasions were observed in 16.3% of the dismantlers. Scars were observed on the skins of 93.6% of the subjects. The prevalence of burns was 23.1%.
Armah et al. (2019) [37]	10	Cross-sectional	140 resident e-waste workers and 60 resident non-e-waste workers, Ghana	60 non-resident non-e-waste workers	NA	Self-reported physical injuries and respiratory symptoms	Generalized linear models (logit, complementary log-log, negative log-log regressions)	Non-e-waste exposed populations are 74% less likely to report eye problems compared with those exposed to e-waste. E-waste workers were 84% more likely to report skin burns compared with non-e-waste workers. Resident e-waste workers reported more breathing difficulty (odds ratio [OR] = 3.30, confidence interval [CI]: 1.733–6.267).
Burns et al. (2019) [39]	48	Cross-sectional	46 e-waste workers, Ghana	None	NA	Injury experience, noise exposures, and stress risk factors	Multivariable Poisson regression	Average injuries per person = 9.9 ± 9.6 (range: 1–40). The majority of injuries were lacerations (65.2%), and the most common injury location was the hand (45.7%). Perceived stress level and perceived noise exposure were associated with a significantly greater number of injuries.
Ohajinwa et al. (2018) [30]	34	Cross-sectional	279 e-waste workers, Nigeria	None	NA	Physical injury patterns	Logistic regression.	Injury prevalence of 38% in 1–2 weeks preceding the study and 68% in the 6-month preceding study. The most common injury type is cuts to the hands and fingers, mainly caused by sharp objects.
Houessionon et al. (2021) [38]	1	Cross-sectional	45 e-waste recyclers, Benin	None	NA	Self-reported injuries	Chi-square test	High prevalence of injuries (88.9%), itchy skin (45.2%), blood in the stool (16.3%), and blood in the urine (14.2%). The number of hours worked per day is associated with blood in urine, itchy skin, and airway obstruction.
Burns et al. (2016) [25]	71	Cross-sectional	57 e-waste workers, Ghana	None	NA	Noise exposures, heart rate, and perceived stress	Mixed effects linear regression model	43.5% of workers had noise exposures that exceeded recommended occupational (85 dBA) and community (70 dBA) noise exposure limits. A 1 dB increase in noise exposure was associated with a 0.17 increase in heart rate (*p* = 0.01).
Carlson et al. (2021) [28]	0	Cross-sectional	58 e-waste workers, Ghana	None	Pb, Cd, total Hg, Se, Mn, Cu, Fe, Zn, and As.	Noise-induced hearing loss (NIHL)	Linear regression models	Audiometric notches indicative of noise-induced hearing loss (NIHL) = 60%, high noise while working = 86%, daily average noise levels range = 74.4–90.0 dBA, and living at Agbogbloshie is associated with worse hearing thresholds at 4 and 6 kHz.
Kêdoté et al. (2022) [32]	1	Cross-sectional	740 e-waste workers, 5 West African countries: Benin, Burkina Faso, Ivory Coast, Mali, and Senegal	None	NA	Perceived stress	Multivariate logistic regression	Prevalence of perceived = 76.76%. Factors associated with stress included insufficient income, number of working days per week, violence at work, and the interference of work with family responsibilities or leisure.
Amoabeng Nti et al. (2020) [21]	41	Longitudinal study	142 e-waste workers, Ghana	65 non-e-waste workers	PM (_2.5_, _2.5–10_, and _10_ µm)	Lung function parameters (FVC, FEV1, FEV1/FVC, PEF, and FEF 25–75)	Random effects models	Median PM concentrations (_2.5_, _2.5–10_, and _10_ µm) were all above the WHO ambient air standards across the 3 study waves. A 10 µg increase in PM (_2.5_, _2.5–10_ and _10_ µm) was associated with decreases in PEF and FEF 25–75 by 13.3% (β = −3.133; 95% CI: −0.243, −0.022) and 26.6% (β = −0.266; 95% CI: −0.437, 0.094), respectively.
Faomowe Foko et al. (2021) [31]	0	Cross-sectional	178 e-waste workers, Senegal	None	NA	Respiratory health	Proportions	Spirometry examination revealed 37.11% cases of asthma, 21.65% cases of COPD-like syndrome, and 7.22% cases of restrictive syndromes.
Igharo et al. (2020) [40]	3	Cross-sectional	63 e-waste workers, Nigeria	41 non-e-waste workers	Pb and Cd	Cardiovascular health	Student’s t-test and Pearson’s correlation	Blood Pb µmol/L, mean ± SD = 0.05 ± 0.006 vs. 0.03 ± 0.03, and Cd nmol/L = 103.20 ± 11.98 vs. 54.65 ± 8.47 (P_all_ < 0.05). Total cholesterol, LDL cholesterol, AC, CRI-1, and CRI-11 significantly increased in the e-waste exposed participants compared to the unexposed group.
Amoabeng Nti et al. (2021) [35]	2	Longitudinal study	142 e-waste workers, Ghana	65 non-e-waste workers	PM (_2.5_, _2.5–10_, and _10_ µm)	Cardiovascular function (heart rate (HR), HRV and blood pressure)	Random effects models	A 10 μg/m^3^ increase in the concentrations of PM_2.5_, PM_10–2.5_, and PM_10_ in personal air was associated with reduced HRV indices and increased resting HR. A 10 μg/m^3^ per interquartile (IQR) increase in PM_10–2.5_ and PM_10_ decreased SDNN by 11% (95% CI: −0.002–0.000; *p* = 0.187) and 34% (95% CI: −0.002–0.001; *p* = 0.035), respectively.
Takyi et al. (2020) [36]	10	Longitudinal study	142 e-waste workers, Ghana	65 non-e-waste workers	Breathing zone PM_2.5_	Cardiovascular indices: systolic BP (SBP), diastolic BP (DBP), and pulse pressure (PP)	Random effects models	Exposure to 1 μg/m^3^ of PM_2.5_ was associated with an increased heart rate (HR) among e-waste recyclers.
Igaro et al. (2015) [41]	2	Cross-sectional	63 e-waste workers, Nigeria	41 non-e-waste workers	NA	Liver damage	Student’s *t*-test	Significantly raised activities of enzymatic biomarkers of liver damage (ALT, AST, ALP, and GGT) in the e-waste group compared with the unexposed participants.
Alabi et al. (2020) [16]	22	Cross-sectional	95 e-waste workers, Nigeria	103 non-e-waste workers	Pb, Ni, Cd, and Cr	DNA damage (micronuclei, binucleated cells, pycnosis)	Spearman correlation	Blood Pb μg/L (median = 19.55, vs. 1.50; *p* = 0.01), Cd μg/L (median = 1.43, vs. 0.18; *p* = 0.05), Cr μg/L (median = 2.31, vs. 0.02; *p* = 0.01), and Ni μg/L (median = 1.05, vs. 0.01; *p* = 0.01). Micronuclei: mean (168.04 vs. 3.23; *p* < 0.01); binucleated cells: (42.20 vs. 0.08; *p* < 0.01); pycnosis: (26.02 vs. 0.00; *p* < 0.01).
Issah et al. (2022) [42]	0	Cross-sectional	100 e-waste workers, Ghana	51 non-e-waste workers	Pb, Cd, Mn, Se, and Zn	Global (LINE-1) DNA methylation	Multivariable linear regression with robust standard errors	Blood Pb, GM (95% CI) = 79.6 (71.9, 88.1) vs. 37.7 (33.8, 42.0), Cd = 147.7 (140.1, 155.8) vs. 190.6 (179.4, 200.7). Blood Zn was associated with a significant decrement in global DNA methylation levels (βZn = −0.912; 95% CI, −1.512, −0.306; *p* = 0.003).

FVC = forced vital capacity; FEV1 = forced expiratory volume in one second; FEV1/FVC = ratio of forced expiratory volume in one second and forced vital capacity; PEF = peaked expiratory flow; FEF25–75 = forced expiratory flow 25–75. COPD = chronic obstructive pulmonary disease; Pb = lead; Cd = cadmium; PM = particulate matter; HR = heart rate; HRV = heart rate variability; LDL = low density lipoprotein; AC = atherogenic coefficient; CRI-1= Castelli risk index 1; CRI-11= Castelli risk index 11. ALT = alanine aminotransferase; AST = aspartate aminotransferase; ALP = alkaline phosphatase; GGT = gamma glutamyltransferase; LINE-1 = long interspersed nucleotide element 1; GM = geometric mean; Cr = chromium; Ni = nickel.

## Data Availability

Not applicable.

## Supplementary Materials

The following supporting information can be downloaded at: https://www.mdpi.com/article/10.3390/ijerph192114278/s1. Table S1: PRISMA 2020 checklist, Table S2: Search strategies and results from different electronic databases, Table S3: Methodological quality of included cross-sectional studies, Table S4: Methodological quality of included cohort studies, Table S5: List of excluded studies at full-text screening stage with brief reasons, Table S6: JBI Critical Appraisal Checklist for Analytical Cross Sectional Studies, Table S7: JBI Critical Appraisal Checklist for Cohort Studies. References [62,63,64,65,66,67,68,69,70] are cited in the supplementary materials.
