# Supplementary material for: Health Risks Associated with Informal Electronic Waste Recycling in Africa: A Systematic Review
Source: Int J Environ Res Public Health. 2022 Nov 1;19(21):14278. doi: 10.3390/ijerph192114278 (PMC9655142; doi:10.3390/ijerph192114278)
Supplement: Supplementary file 1 [file ijerph-19-14278-s001.zip › ijerph-1882506-supplementary.pdf]

Health Risks Associated with Informal Electronic Waste Recycling in Africa: A Systematic Review

Ibrahim Issah [1,*], John Arko-Mensah [1], Thomas P. Agyekum [1,2], Duah Dwomoh [3] and Julius N. Fobil [1]

[1]Department of Biological, Environmental and Occupational Health Sciences, School of Public Health, College of Health Sciences, University of Ghana, Legon – Accra, Ghana

[2]Department of Occupational and Environmental Health and Safety, School of Public Health, College of Health Sciences, Kwame Nkrumah University of Science and Technology, Kumasi, Ghana

[3]Department of Biostatistics, School of Public Health, College of Health Sciences, University of Ghana, Legon – Accra, Ghana

[*]Correspondence: ibrahimissah111@gmail.com

1. Supplementary Table S1: PRISMA 2020 checklist
2. Supplementary Table S2: Search strategies and results from different electronic databases
3. Supplementary Table S3: Methodological quality of included cross-sectional studies
4. Supplementary Table S4: Methodological quality of included cohort studies
5. Supplementary Table S5: List of excluded studies at full-text screening stage with brief reasons
6. Supplementary Table S6: Joanna Brigg's Institute (JBI) critical appraisal checklist for analytical cross-sectional studies
7. Supplementary Table S7: Joanna Brigg's Institute (JBI) critical appraisal checklist for cohort studies

Supplementary Table S1. PRISMA 2020 checklist

Section and Topic	Item #	Checklist item	Location where item is reported
**TITLE**			
Title	1	Identify the report as a systematic review.	Page 1
**ABSTRACT**			
Abstract	2	See the PRISMA 2020 for Abstracts checklist.	Page 1
**INTRODUCTION**			
Rationale	3	Describe the rationale for the review in the context of existing knowledge.	Page 2-3
Objectives	4	Provide an explicit statement of the objective(s) or question(s) the review addresses.	Page 3
**METHODS**			
Eligibility criteria	5	Specify the inclusion and exclusion criteria for the review and how studies were grouped for the syntheses.	Page 3
Information sources	6	Specify all databases, registers, websites, organisations, reference lists and other sources searched or consulted to identify studies. Specify the date when each source was last searched or consulted.	Page 3-4
Search strategy	7	Present the full search strategies for all databases, registers and websites, including any filters and limits used.	Page 4 Supplementary material (Table 2), page 5
Selection process	8	Specify the methods used to decide whether a study met the inclusion criteria of the review, including how many reviewers screened each record and each report retrieved, whether they worked independently, and if applicable, details of automation tools used in the process.	Page 4
Data collection process	9	Specify the methods used to collect data from reports, including how many reviewers collected data from each report, whether they worked independently, any processes for obtaining or confirming data from study investigators, and if applicable, details of automation tools used in the process.	Page 4
Data items	10a	List and define all outcomes for which data were sought. Specify whether all results that were compatible with each outcome domain in each study were sought (e.g. for all measures, time points, analyses), and if not, the methods used to decide which results to collect.	
	10b	List and define all other variables for which data were sought (e.g. participant and intervention characteristics, funding sources). Describe any assumptions made about any missing or unclear information.	
Study risk of bias assessment	11	Specify the methods used to assess risk of bias in the included studies, including details of the tool(s) used, how many reviewers assessed each study and whether they worked independently, and if applicable, details of automation tools used in the process.	Page 4-5
Effect measures	12	Specify for each outcome the effect measure(s) (e.g. risk ratio, mean difference) used in the synthesis or presentation of results.	
Synthesis	13a	Describe the processes used to decide which studies were eligible for each synthesis (e.g. tabulating the study intervention characteristics	

Section and Topic	Item #	Checklist item	Location where item is reported
methods		and comparing against the planned groups for each synthesis (item #5)).	
	13b	Describe any methods required to prepare the data for presentation or synthesis, such as handling of missing summary statistics, or data conversions.	
	13c	Describe any methods used to tabulate or visually display results of individual studies and syntheses.	
	13d	Describe any methods used to synthesize results and provide a rationale for the choice(s). If meta-analysis was performed, describe the model(s), method(s) to identify the presence and extent of statistical heterogeneity, and software package(s) used.	
	13e	Describe any methods used to explore possible causes of heterogeneity among study results (e.g. subgroup analysis, meta-regression).	
	13f	Describe any sensitivity analyses conducted to assess robustness of the synthesized results.	
Reporting bias assessment	14	Describe any methods used to assess risk of bias due to missing results in a synthesis (arising from reporting biases).	
Certainty assessment	15	Describe any methods used to assess certainty (or confidence) in the body of evidence for an outcome.	
**RESULTS**			
Study selection	16a	Describe the results of the search and selection process, from the number of records identified in the search to the number of studies included in the review, ideally using a flow diagram.	Page 6
	16b	Cite studies that might appear to meet the inclusion criteria, but which were excluded, and explain why they were excluded.	Supplementary material (Table 5) page 7
Study characteristics	17	Cite each included study and present its characteristics.	Page 6-12
Risk of bias in studies	18	Present assessments of risk of bias for each included study.	Page 13 and Supplementary material (Tables 3 and 4) page 6
Results of individual studies	19	For all outcomes, present, for each study: (a) summary statistics for each group (where appropriate) and (b) an effect estimate and its precision (e.g. confidence/credible interval), ideally using structured tables or plots.	Table 1, page 8-12
Results of syntheses	20a	For each synthesis, briefly summarise the characteristics and risk of bias among contributing studies.	Page 13-16
	20b	Present results of all statistical syntheses conducted. If meta-analysis was done, present for each the summary estimate and its precision (e.g. confidence/credible interval) and measures of statistical heterogeneity. If comparing groups, describe the direction of the effect.	
	20c	Present results of all investigations of possible causes of heterogeneity among study results.	
	20d	Present results of all sensitivity analyses conducted to assess the robustness of the synthesized results.	
Reporting biases	21	Present assessments of risk of bias due to missing results (arising from reporting biases) for each synthesis assessed.	

Section and Topic	Item #	Checklist item	Location where item is reported
Certainty of evidence	22	Present assessments of certainty (or confidence) in the body of evidence for each outcome assessed.	
**DISCUSSION**			
Discussion	23a	Provide a general interpretation of the results in the context of other evidence.	Page 17-18
	23b	Discuss any limitations of the evidence included in the review.	Page 18
	23c	Discuss any limitations of the review processes used.	
	23d	Discuss implications of the results for practice, policy, and future research.	Page 18-19
**OTHER INFORMATION**			
Registration and protocol	24a	Provide registration information for the review, including register name and registration number, or state that the review was not registered.	Page 20
	24b	Indicate where the review protocol can be accessed, or state that a protocol was not prepared.	Page 20
	24c	Describe and explain any amendments to information provided at registration or in the protocol.	
Support	25	Describe sources of financial or non-financial support for the review, and the role of the funders or sponsors in the review.	
Competing interests	26	Declare any competing interests of review authors.	Page 20
Availability of data, code and other materials	27	Report which of the following are publicly available and where they can be found: template data collection forms; data extracted from included studies; data used for all analyses; analytic code; any other materials used in the review.	

From: Page MJ, McKenzie JE, Bossuyt PM, Boutron I, Hoffmann TC, Mulrow CD, et al. The PRISMA 2020 statement: an updated guideline for reporting systematic reviews. BMJ 2021;372:n71. doi: 10.1136/bmj.n71

For more information, visit: http://www.prisma-statement.org/

Supplementary Table S2. Search strategies and results from different electronic databases

SCOPUS	
**Search**	**Query**
1	("Electronic waste" OR "E-waste" OR "Waste electrical and electronic equipment" OR "WEEE")
2	("Health" OR "Respiratory health" OR "injuries" OR "Cardiovascular health" OR "heart rate variability" OR "blood pressure" OR "DNA" OR "musculoskeletal disorders" OR "stress" OR "hearing disorders")
3	("Africa" OR "Sub-Saharan Africa" OR "developing countries" OR "Cameroon" OR "Chad" OR "Congo" OR "Democratic Republic of Congo" OR "Congo, Demographic Republic" OR "Congo, Republic" OR "Equatorial Guinea" OR "Gabon" OR "Burundi" OR "Djibouti" OR "Eritrea" OR "Ethiopia" OR "Kenya" OR "Rwanda" OR "Somalia" OR "Sudan" OR "Tanzania" OR "Uganda" OR "Angola" OR "Botswana" OR "Lesotho" OR "Malawi" OR "Mozambique" OR "Namibia" OR "Swaziland" OR "Zambia" OR "Zimbabwe" OR "Benin" OR "Burkina Faso" OR "Cape Verde" OR "Cote D'ivoire" OR "Gambia" OR "Ghana" OR "Guinea" OR "Guinea-Bissau" OR "Liberia" OR "Mali" OR "Mauritania" OR "Niger" OR "Nigeria" OR "Senegal" OR "Sierra Leone" OR "Togo" OR "South Sudan" OR "Madagascar" OR "Comoros" OR "Mauritius" OR "Sao Tome and Principe" OR "Seychelles" OR "South Africa" OR “Algeria” OR “Egypt” OR “Libya” OR “Morocco” OR “Tunisia”)
4	1 AND 2 AND 3 = 428
Limiters	English Language<br>Academic/Peer reviewed Journals
**MEDLINE via EBSCOhost**	
**Search**	**Query**
1	("Electronic waste" OR "E-waste" OR "Waste electrical and electronic equipment" OR "WEEE")
2	("Health" OR "Respiratory health" OR "injuries" OR "Cardiovascular health" OR "heart rate variability" OR "blood pressure" OR "DNA" OR "musculoskeletal disorders" OR "stress" OR "hearing disorders")
3	("Africa" OR "Sub-Saharan Africa" OR "developing countries" OR "Cameroon" OR "Chad" OR "Congo" OR "Democratic Republic of Congo" OR "Congo, Demographic Republic" OR "Congo, Republic" OR "Equatorial Guinea" OR "Gabon" OR "Burundi" OR "Djibouti" OR "Eritrea" OR "Ethiopia" OR "Kenya" OR "Rwanda" OR "Somalia" OR "Sudan" OR "Tanzania" OR "Uganda" OR "Angola" OR "Botswana" OR "Lesotho" OR "Malawi" OR "Mozambique" OR "Namibia" OR "Swaziland" OR "Zambia" OR "Zimbabwe" OR "Benin" OR "Burkina Faso" OR "Cape Verde" OR "Cote D'ivoire" OR "Gambia" OR "Ghana" OR "Guinea" OR "Guinea-Bissau" OR "Liberia" OR "Mali" OR "Mauritania" OR "Niger" OR "Nigeria" OR "Senegal" OR "Sierra Leone" OR "Togo" OR "South Sudan" OR "Madagascar" OR "Comoros" OR "Mauritius" OR "Sao Tome and Principe" OR "Seychelles" OR "South Africa" OR “Algeria” OR “Egypt” OR “Libya” OR “Morocco” OR “Tunisia”)
4	1 AND 2 AND 3 = 295
Limiters	English Language<br>Academic/Peer reviewed Journals

Supplementary Table S3. methodological quality of included cross-sectional studies

Author(s)	Cross-sectional studies								
	Q1	Q2	Q3	Q4	Q5	Q6	Q7	Q8	Score
Acquah et al (2021) [1]	Yes	Yes	No	NA	Yes	Yes	No	Yes	5
Adusei et al (2020) [2]	Yes	Yes	Yes	NA	No	No	Yes	Yes	5
Armah et al (2019) [3]	Yes	Yes	Yes	NA	Yes	Yes	No	Yes	6
Alabi et al (2020) [4]	Yes	Yes	Yes	NA	Yes	Yes	Yes	Yes	7
Burns et al (2019) [5]	Yes	No	Yes	NA	Yes	Yes	No	Yes	5
Burns et al (2016) [6]	Yes	Yes	Yes	NA	Yes	Yes	Yes	Yes	7
Carlson et al (2021) [7]	Yes	Yes	Yes	NA	Yes	Yes	Yes	Yes	7
Faomowe Foko et al (2021) [8]	Yes	Yes	No	NA	Yes	Yes	Yes	Yes	6
Fischer et al (2020) [9]	Yes	No	No	NA	No	No	No	Yes	2
Igaro et al (2015) [10]	Yes	Yes	No	NA	No	No	Yes	Yes	4
Igharo et al (2020) [11]	Yes	Yes	No	NA	No	No	Yes	Yes	4
Issah et al (2022) [12]	Yes	Yes	Yes	NA	Yes	Yes	Yes	Yes	7
Kêdoté et al (2022) [13]	Yes	Yes	No	NA	Yes	Yes	Yes	Yes	6
Ohajinwa et al (2018) [14]	Yes	Yes	No	NA	Yes	Yes	No	Yes	5
Houessionon et al (2021) [15]	Yes	Yes	No	NA	Yes	Yes	No	Yes	5

Q=question, NA=Not applicable

Supplementary Table S4. methodological quality of included cohort studies

Author(s)	Cohort studies											
	Q1	Q2	Q3	Q4	Q5	Q6	Q7	Q8	Q9	Q10	Q11	Score
Amoabeng Nti et al (2020) [16]	Yes	Yes	Yes	Yes	Yes	NC	Yes	No	Yes	Yes	Yes	9
Amoabeng Nti et al (2021) [17]	Yes	Yes	Yes	Yes	Yes	NC	Yes	No	Yes	Yes	Yes	9
Takyi et al (2020) [18]	Yes	Yes	Yes	Yes	Yes	NC	Yes	Yes	Yes	Yes	Yes	10

Q=question; NC=Not clear

Supplementary Table S5. List of excluded studies at full-text screening stage with brief reasons

SN	Author(year)	Country	Title	Article type	Reason for exclusion
1	Acquah et al (2021) [19]	Ghana	Work-Related Exposures and Musculoskeletal Disorder Symptoms among Informal E-Waste Recyclers at Agbogbloshie, Ghana	Article	Book chapter
2	Acquah et al (2021) [20]	Ghana	Comparison of ergonomic risk factors and work-related musculoskeletal disorders among dismantler and burners of electronic waste in Agbogbloshie, Accra Ghana	Article	Shared the same population and outcome with another study already included
3	Acquah et al (2021) [21]	Ghana	A preliminary assessment of physical work exposures among electronic waste workers at Agbogbloshie, Accra Ghana	Article	Did not report human health outcome

4	Fobil et al (2021) [22]	Ghana	Occupational and Environmental Health Effects of Informal Electronic Waste Recycling – A Focus on Agbogbloshie, Ghana	Review	A review
5	Matovu et al (2019) [23]	Uganda	Polybrominated diphenyl ethers in mothers' breast milk and associated health risk to nursing infants in Uganda	Article	Did not report human health outcome
6	Ohajinwa et al (2019) [24]	Nigeria	Health risks of polybrominated diphenyl ethers (PBDEs) and metals at informal electronic waste recycling sites	Article	Did not report human health outcome
7	D'Souza et al (2021) [25]	Ghana	Musculoskeletal Disorders in Unstructured, Unregulated Work: Assessment Methods and Injuries	Article	Book chapter
8	Issah et al (2021) [26]	Ghana	Association between global DNA methylation (LINE-1) and occupational particulate matter exposure among informal electronic-waste recyclers in Ghana	Article	Shared the same population and outcome with another study already included
9	Issah et al (2021) [27]	Ghana	Global DNA (LINE-1) methylation is associated with lead exposure and certain job tasks performed by electronic waste workers	Article	Shared the same population and outcome with another study already included
10	Fischer et al (2020) [9]	Ghana	Health consequences for E-waste workers and bystanders—a comparative cross-sectional study	Article	Poor methodological quality assessment score

Supplementary Table S6.

JBI Critical Appraisal Checklist for Analytical Cross Sectional Studies

	Yes	No	Unclear	Not applicable
1. Were the criteria for inclusion in the sample clearly defined?	☐	☐	☐	☐
2. Were the study subjects and the setting described in detail?	☐	☐	☐	☐
3. Was the exposure measured in a valid and reliable way?	☐	☐	☐	☐
4. Were objective, standard criteria used for measurement of the condition?	☐	☐	☐	☐
5. Were confounding factors identified?	☐	☐	☐	☐
6. Were strategies to deal with confounding factors stated?	☐	☐	☐	☐
7. Were the outcomes measured in a valid and reliable way?	☐	☐	☐	☐
8. Was appropriate statistical analysis used?	☐	☐	☐	☐

Overall appraisal: Include ☐ Exclude ☐ Seek further info ☐

Comments (Including reason for exclusion)

Supplementary Table S7.
JBI Critical Appraisal Checklist for Cohort Studies

	Yes	No	Unclear	Not applicable
1. Were the two groups similar and recruited from the same population?	□	□	□	□
2. Were the exposures measured similarly to assign people to both exposed and unexposed groups?	□	□	□	□
3. Was the exposure measured in a valid and reliable way?	□	□	□	□
4. Were confounding factors identified?	□	□	□	□
5. Were strategies to deal with confounding factors stated?	□	□	□	□
6. Were the groups/participants free of the outcome at the start of the study (or at the moment of exposure)?	□	□	□	□
7. Were the outcomes measured in a valid and reliable way?	□	□	□	□
8. Was the follow up time reported and sufficient to be long enough for outcomes to occur?	□	□	□	□
9. Was follow up complete, and if not, were the reasons to loss to follow up described and explored?	□	□	□	□
10. Were strategies to address incomplete follow up utilized?	□	□	□	□
11. Was appropriate statistical analysis used?	□	□	□	□

Overall appraisal: Include □ Exclude □ Seek further info □

Comments (Including reason for exclusion)

References

1. Acquah, A A, D'souza, C, Martin, B J, Arko-Mensah, J, Dwomoh, D, Nti, A A A, et al., *Musculoskeletal Disorder Symptoms among Workers at an Informal Electronic-Waste Recycling Site in Agbogbloshie, Ghana.* International Journal of Environmental Research and Public Health, 2021. **18**(4): p. 1-20.
2. Adusei, A, Arko-Mensah, J, Dzodzomenyo, M, Stephens, J, Amoabeng, A, Waldschmidt, S, et al., *Spatiality in Health: The Distribution of Health Conditions Associated with Electronic Waste Processing Activities at Agbogbloshie, Accra.* Annals of Global Health, 2020. **86**(1).
3. Armah, F A, Quansah, R, Yawson, D O, and Abdul Kadir, L, *Assessment of Self-Reported Adverse Health Outcomes of Electronic Waste Workers Exposed to Xenobiotics in Ghana.* Environmental Justice, 2019. **12**(2): p. 69-84.
4. Alabi, O A, Adeoluwa, Y M, and Bakare, A A, *Elevated Serum Pb, Ni, Cd, and Cr Levels and DNA Damage in Exfoliated Buccal Cells of Teenage Scavengers at a Major Electronic Waste Dumpsite in Lagos, Nigeria.* Biological Trace Element Research, 2020. **194**(1): p. 24-33.
5. Burns, K N, Sayler, S K, and Neitzel, R L, *Stress, Health, Noise Exposures, and Injuries among Electronic Waste Recycling Workers in Ghana.* Journal of Occupational Medicine and Toxicology, 2019. **14**(1).
6. Burns, K N, Sun, K, Fobil, J N, and Neitzel, R L, *Heart Rate, Stress, and Occupational Noise Exposure among Electronic Waste Recycling Workers.* International Journal of Environmental Research and Public Health, 2016. **13**(1).
7. Carlson, K, Basu, N, Fobil, J N, and Neitzel, R L, *Metal Exposures, Noise Exposures and Audiometry from E-Waste Workers in Agbogbloshie, Ghana.* International Journal of Environmental Research and Public Health, 2021. **18**(18).
8. Faomowe Foko, R, Mbodji, M, Kêdote, M, Diaw, M, Touré, M, Bah, F, et al., *Chemical Risk and Respiratory Health of Waste Electrical and Electronic Equipment (Weee) Handlers in Dakar (Senegal).* Archives des Maladies Professionnelles et de l'Environnement, 2021. **82**(4): p. 438-448.
9. Fischer, D, Seidu, F, Yang, J, Felten, M K, Garus, C, Kraus, T, et al., *Health Consequences for E-Waste Workers and Bystanders—a Comparative Cross-Sectional Study.* International Journal of Environmental Research and Public Health, 2020. **17**(5).
10. Igaro, O G, Anetor, J I, Osibanjo, O O, Osadolor, H B, Idomeh, F A, Igiewe, W O, et al., *Liver Damage Risk Assessment Study in Workers Occupationally Exposed to E-Waste in Benin City, South-South Nigeria.* Journal of Chemical Health Risks, 2015. **5**(3): p. 155-166.
11. Igharo, O G, Akinfenwa, Y, Isara, A R, Idomeh, F A, Nwobi, L N, Anetor, J I, et al., *Lipid Profile and Atherogenic Indices in Nigerians Occupationally Exposed to E-Waste: A Cardiovascular Risk Assessment Study.* Maedica - a Journal of Clinical Medicine, 2020. **15**(2): p. 196-205.
12. Issah, I, Arko-Mensah, J, Rozek, L S, Zarins, K R, Dwomoh, D, Agyekum, T P, et al., *Association between Toxic and Essential Metals in Blood and Global DNA Methylation among Electronic Waste Workers in Agbogbloshie, Ghana.* Environmental Science and Pollution Research, 2022: p. 1-11.
13. Kêdoté, N M, Sopoh, G E, Tobada, S B, Darboux, A J, Fonton, P, Sanon Lompo, M S, et al., *Perceived Stress at Work and Associated Factors among E-Waste Workers in French-Speaking West Africa.* International Journal of Environmental Research and Public Health, 2022. **19**(2).
14. Ohajinwa, C M, Van Bodegom, P M, Vijver, M G, Olumide, A O, Osibanjo, O, and Peijnenburg, W J G M, *Prevalence and Injury Patterns among Electronic Waste Workers in the Informal Sector in Nigeria.* Injury Prevention, 2018. **24**(3): p. 185-192.
15. Houessionon, M K, Basu, N, Bouland, C, Kedote, N M, Fayomi, B, Fobil, N J, et al., *Knowledge, Practices, and Environmental and Occupational Health Risks Associated with Electronic Waste Recycling in Cotonou, Benin.* Occupational Diseases and Environmental Medicine, 2021. **9**(2): p. 33-48.

16. Amoabeng Nti, A A, Arko-Mensah, J, Botwe, P K, Dwomoh, D, Kwarteng, L, Takyi, S A, et al., *Effect of Particulate Matter Exposure on Respiratory Health of E-Waste Workers at Agbogbloshie, Accra, Ghana.* International Journal of Environmental Research and Public Health, 2020. **17**(9).
17. Amoabeng Nti, A A, Robins, T G, Mensah, J A, Dwomoh, D, Kwarteng, L, Takyi, S A, et al., *Personal Exposure to Particulate Matter and Heart Rate Variability among Informal Electronic Waste Workers at Agbogbloshie: A Longitudinal Study.* BMC Public Health, 2021. **21**(1).
18. Takyi, S A, Basu, N, Arko-Mensah, J, Botwe, P, Amoabeng Nti, A A, Kwarteng, L, et al., *Micronutrient-Rich Dietary Intake Is Associated with a Reduction in the Effects of Particulate Matter on Blood Pressure among Electronic Waste Recyclers at Agbogbloshie, Ghana.* BMC Public Health, 2020. **20**(1).
19. Acquah, A A, D'Souza, C, Martin, B, Arko-Mensah, J, Quakyi, I A, Basu, N, et al., *Work-Related Exposures and Musculoskeletal Disorder Symptoms among Informal E-Waste Recyclers at Agbogbloshie, Ghana.* Proceedings of the 21st Congress of the International Ergonomics Association (IEA 2021). Volume IV, Healthcare and Healthy Work. International Ergonomics Association. Congress, 2021. **222**: p. 677-681.
20. Acquah, A A, D'Souza, C, Martin, B J, Arko-Mensah, J, Basu, N, Quakyi, I A, et al., *Comparison of Ergonomic Risk Factors and Work-Related Musculoskeletal Disorders among Dismantler and Burners of Electronic Waste in Agbogbloshie, Accra Ghana.* Proceedings of the Human Factors and Ergonomics Society ... Annual Meeting. Human Factors and Ergonomics Society. Annual meeting, 2021. **65**(1): p. 715-719.
21. Acquah, A A, D'Souza, C, Martin, B J, Arko-Mensah, J, Botwe, P K, Tettey, P, et al., *A Preliminary Assessment of Physical Work Exposures among Electronic Waste Workers at Agbogbloshie, Accra Ghana.* International Journal of Industrial Ergonomics, 2021. **82**.
22. Fobil, J, Abotsi, P, Acquah, A A, Arko-Mensah, J, D'Souza, C, and Martin, B, *Occupational and Environmental Health Effects of Informal Electronic Waste Recycling – a Focus on Agbogbloshie, Ghana*, in *Lecture Notes in Networks and Systems*. 2021. p. 746-752.
23. Matovu, H, Sillanpää, M, and Ssebugere, P, *Polybrominated Diphenyl Ethers in Mothers' Breast Milk and Associated Health Risk to Nursing Infants in Uganda.* Science of the Total Environment, 2019. **692**: p. 1106-1115.
24. Ohajinwa, C M, van Bodegom, P M, Osibanjo, O, Xie, Q, Chen, J, Vijver, M G, et al., *Health Risks of Polybrominated Diphenyl Ethers (Pbdes) and Metals at Informal Electronic Waste Recycling Sites.* International Journal of Environmental Research and Public Health, 2019. **16**(6).
25. D'Souza, C, Martin, B, Fobil, J N, Todd, A, and Gomes, J O, *Musculoskeletal Disorders in Unstructured, Unregulated Work: Assessment Methods and Injuries.* Proceedings of the 21st Congress of the International Ergonomics Association (IEA 2021). Volume IV, Healthcare and Healthy Work. International Ergonomics Association. Congress, 2021. **222**: p. 720-727.
26. Issah, I, Arko-Mensah, J, Rozek, L S, Rentschler, K, Agyekum, T P, Dwumoh, D, et al., *Association between Global DNA Methylation (Line-1) and Occupational Particulate Matter Exposure among Informal Electronic-Waste Recyclers in Ghana.* International Journal of Environmental Health Research, 2021.
27. Issah, I, Arko-Mensah, J, Rozek, L S, Zarins, K R, Agyekum, T P, Dwomoh, D, et al., *Global DNA (Line-1) Methylation Is Associated with Lead Exposure and Certain Job Tasks Performed by Electronic Waste Workers.* International Archives of Occupational and Environmental Health, 2021. **94**(8): p. 1931-1944.
